# Prognostic Significance of Regional/Systemic Metabolic Parameters on ^18^F-FDG PET in Pulmonary Lymphoepithelioma-Like Carcinoma

**DOI:** 10.3389/fonc.2021.675961

**Published:** 2021-06-17

**Authors:** Yongjiang Li, Wenbiao Zhang, Hu Zhang, Junfeng Hu, Chao Zhou, Xu Zhang, Wei Fan

**Affiliations:** ^1^ Department of Nuclear Medicine, Sun Yat-Sen University Cancer Center, Sun Yat-Sen University, Guangzhou, China; ^2^ Department of Medical Imaging, Sun Yat-Sen University Cancer Center, Sun Yat-Sen University, Guangzhou, China

**Keywords:** PET, 18F-fluorodeoxyglucose-PET/CT, prognostic significance, metabolic tumor volume (MTV), total lesion glycolysis (TLG)

## Abstract

**Background:**

Pulmonary lymphoepithelioma-like carcinoma (LELC) is a rare subtype of lung cancer with less than 700 cases being reported in the literature, and no specialized treatment guidelines have been established. The prognostic significance of metabolic parameters on ^18^F-FDG PET in pulmonary LELC still remains unknown.

**Methods:**

From July 2011 to September 2020, 76 pulmonary LELC patients undergoing pre-treatment ^18^F-FDG PET imaging were enrolled, and PET parameters including maximum standard uptake value (SUVmax), metabolic tumor volume (MTV), and total lesion glycolysis (TLG) were calculated. In addition, whole-body tumor burdens were categorized into primary tumor lesion (PRL), thoracic lymph-node lesion (TRLN), and distant metastasis (DM) for respective metabolic parameters acquisition. ROC curves were generated to evaluate the predictive performance of the PET parameters, and correlations between tumor burdens of the different regional lesions were analyzed using linear correlation analysis. The prognostic significance for progression-free survival (PFS) and overall survival (OS) was assessed using univariate and multivariate survival analyses.

**Results:**

Tumor stage, pre-/post-treatment serum EBV-DNA copies, SUVmax (cutoff 17.5), MTV, and TLG were significantly associated with PFS and OS in univariate analysis. MTV and TLG (AUC = 0.862 and 0.857, respectively) showed significantly higher predictive value than SUVmax (AUC = 0.754) and remained independent prognostic indicators for PFS in multivariate analysis (P = 0.026 and 0.019, respectively). Besides, non-colinearity was detected between metabolic burdens of the different regional lesions. MTV_-PRL_, MTV_-DM,_ TLG_-PRL_, and TLG_-DM_ were identified to be independent prognostic factors for PFS and OS, whereas MTV_-TRLN_ and TLG_-TRLN_ were not.

**Conclusion:**

The study demonstrated that MTV and TLG had independent prognostic significance for pulmonary LELC, which supported the incorporation of ^18^F-FDG PET imaging into clinical treatment protocols for pulmonary LELC and implied multi-disciplinary cooperation for primary and distant metastatic lesions to further improve prognosis.

## Introduction

Pulmonary lymphoepithelioma-like carcinoma (LELC) is a rare and unique subtype of lung cancer that histologically resembles undifferentiated nasopharyngeal carcinoma (NPC) (1, 2). Etiologically, pulmonary LELC is recognized to be closely associated with Epstein–Barr virus (EBV) infection, and less than 700 cases have been reported in the literature due to its rarity. The disease is more prevalent in Asian ethnicity and tends to occur in relatively young and middle-aged individuals irrespective of smoking status and sex (3). The 2015 World Health Organization Classification of Lung Tumors classified pulmonary LELC under the category of “other and unclassified carcinomas” (4). As the standard protocol has not been established because of the lack of clinical trials, the main treatment principle for pulmonary LELC refers to non-small-cell lung cancer (NSCLC) with a more favorable prognosis being achieved. For patients at an early stage, radical surgery with or without chemotherapy remains the primary treatment which results in a 5-year overall survival (OS) rate of about 80% (5, 6). Cisplatin-based chemotherapy or chemo-radiotherapy is the general option for patients at advanced stage, with an expected 5-year OS of 60% (6, 7). Our recent studies have shown that (I) the genomic landscape of pulmonary LELC is distinctive to common histological types of NSCLC, with most of the classic lung cancer driver mutations being undetected but significantly enriched in epigenetic regulator mutations (8); and (II) chemotherapy regimens of gemcitabine plus platinum (AP) achieved the best response rate and could serve as the first-line chemotherapy protocol for pulmonary LELC, which is also distinctive to other NSCLCs (9).

The National Comprehensive Cancer Network recommends the utility of ^18^F-FDG PET as routine examination for NSCLC patients because of its superiority in disease staging, therapeutic response evaluation and outcome prediction (10, 11). In comparison, the clinical and prognostic significance of PET parameters in pulmonary LELC still largely remains nuclear. The first case of pulmonary LELC manifestation on PET imaging was reported in 2015 (12). Until now, only one retrospective study compared two cohorts of pulmonary LELC patients undergoing pre-treatment ^18^F-FDG PET examination (n = 41) or not (n = 30) and found that the patients undergoing pre-treatment PET imaging tended to have better prognosis, mainly due to more precise identification of both intra- and extra-thoracic lesions that ultimately led to more accurate disease staging and treatment option selection (13). However, limited by the sample size, the associations between SUVmax and the patients’ outcomes were not investigated, and further volume-based PET parameters were also not analyzed. The prognostic impact of metabolic PET parameters in pulmonary LELC needs to be clarified.

**Figure 1:**
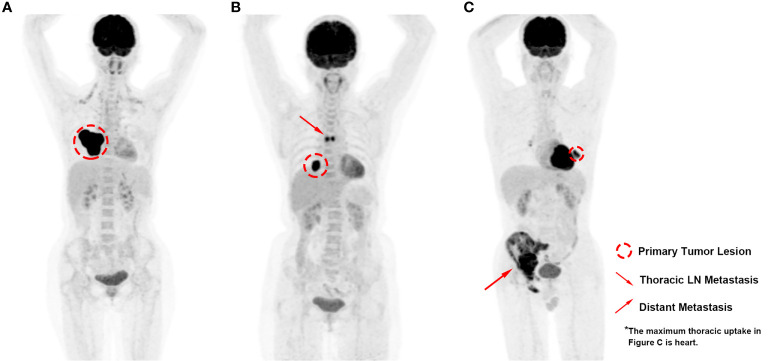
PET images of pulmonary LELC patients with various lesion distribution patterns. **(A)** High metabolic volume of primary lesion without regional or distant metastasis; **(B)** Moderate metabolic volume of primary lesion with moderate metabolic volume of thoracic lymph-node metastasis; **(C)** Low metabolic volume of primary lesion with high metabolic volume of distant metastasis.

Based on the premises, we conducted the current study to specifically investigate the prognostic and clinical significance of metabolic PET parameters in pulmonary LELC. During the retrospective inspection of the PET images, we noticed that the distribution of lesions varied among the patients (**Figure 1**), which indicated that the measurement of only one regional lesion (either primary or metastatic lesion) could generate bias, and raised the question as to what extent of each region of lesion dominated the patients’ prognosis most. Hence, based on the manifestation features of the patients’ PET imaging and the biological behaviors of pulmonary LELC, we classified the tumor lesions into primary tumor lesion (PRL), thoracic lymph-node lesion (TRLN), and distant metastasis (DM) for respective metabolic parameter acquisition and combined them together to generate the whole body tumor burden. In this way, the significance of PET imaging for pulmonary LELC patients could be more comprehensively analyzed.

## Materials And Methods

### Patients

The research protocol of the study was examined and approved by the Institutional Review Board and the Medical Ethics Committee of Sun Yat-Sen Cancer Center. From July 2011 to September 2020, a total of 92 patients with pathologically diagnosed LELC who underwent ^18^F-FDG PET/CT scan at our institution were identified through a retrospective review of electronic clinical records. Of them, 76 pulmonary LELC patients who underwent pre-treatment PET/CT imaging with subsequent medical treatment and follow-up records were finally enrolled in the study. In addition to the PET/CT scan, whole clinical work-ups which consisted of medical history taking, physical examination, fiber optic nasopharyngoscopy, and head–neck CT or MRI were also performed to rule out primary NPC. The pathological diagnosis of LELC was in accordance with the World Health Organization criteria, and disease staging was based on the 7^th^ edition of the American Joint Committee on Cancer Staging (AJCC) system for lung cancer. Pathological stage was utilized for the patients who underwent surgery, and clinical stage was used for those without surgery to ensure the accuracy of staging for the entire cohort.

### Acquisition of ^18^F-FDG PET/CT Images

All patients fasted for 6 h before ^18^F-FDG administration, and the patients’ blood glucose level was checked to ensure it was below 200 mg/dl. PET/CT scans were performed using a Biograph mCT Scanner (Siemens Healthcare, Henkestr, Germany) in 65 patients, and a Discovery ST Scanner (GE Healthcare, Waukesha, USA) in 11 patients. Total body scan was conducted 60 min after the ^18^F-FDG injection (0.1 mCi/kg or 3.7 MBq/kg body weight). CT scans of the whole body from skull to mid-thigh were obtained without contrast enhancement in an arm-up position for attenuation correction and fusion (80–200 mAs, 120 kVp, 3 mm slice thickness for the Biograph mCT scanner; and automatic tube current, 140 kVp, 3.75 mm slice thickness for the Discovery ST scanner) and were reconstructed in a 512 × 512 matrix. The subsequent PET scan was conducted in six to eight bed positions with acquisition time of 2 to 3 min per bed position. The PET images were reconstructed with a slice thickness of 2 mm for the Biograph mCT scanner or 3.25mm for the Discovery ST scanner, using the Ordered Subsets Expectation Maximization (OSEM) iterative reconstruction method. Counter correction and cross-calibration of the two scanners were conducted periodically to ensure an optimal quantitative accuracy, and no significant difference at SUV quantitation was observed between the two scanners.

### Analysis of ^18^F-FDG PET/CT Data

Volume-based assessment of the tumor lesions and PET parameter calculation were performed on a dedicated workstation Syngo.via (Siemens Healthcare, Erlangen). The workstation provided a convenient and automatic way to determine boundaries around a lesion using an isocontour threshold method after a region of interest (ROI) was drawn, and the standard uptake values (SUV) of the lesion including SUVmax and SUVmean were generated. SUV value of the ROI was calculated as the following: SUV = [tissue activity concentration (Bq/ml)]/[injected activity (Bq)/weight (mg)]. SUVmax referred to the value of the hottest voxel with the maximum activity; SUVmean referred to the mean SUV value within the lesion boundary. Volume-based parameters including metabolic tumor volume (MTV) and total lesion glycolysis (TLG) were also generated. MTV was calculated with isocontour threshold SUV of 2.5, and TLG was calculated as MTV multiplied by the SUVmean of the lesion.

All PET/CT images were visually assessed by experienced nuclear medicine physicians, and the location and extent of primary and metastatic lesions was determined. Tumor lesions were divided into three categories for parameter calculation (1): primary lung lesion (PL) (2), thoracic LN lesions (TRLN) including hilar LNs, mediastinum LNs, and supraclavicular LNs if positive, and (3) metastatic lesions (MLs) including bone metastasis, liver metastasis, distant LNs metastasis, and parietal pleura/thoracic wall metastasis. For each category of tumor lesion, the standard uptake and volumetric parameters were calculated individually, and the whole body tumor lesion (WB) referred to the sum of these lesions, of which the SUVmax and SUVpeak equaled to the maximum value, and the MTV and TLG were the sum of all lesions.

### Patients’ Treatment and Follow-Up

Surgery was routinely considered for the stage I patients, and surgery with neoadjuvant/adjuvant chemotherapy (either with or without radiotherapy) was done for the patients with stage II or operable stage III disease. Patients with inoperable stage III–IV disease were treated with chemotherapy or radiochemotherapy. CT, MRI, and/or PET/CT imaging were performed for the post-therapeutic disease status evaluation, and the patients were followed up every 3 months for the first 2 years, and biannually thereafter unless a specific clinical event emerged. The primary endpoint was progression-free survival (PFS), and the secondary endpoint was overall survival (OS) of the patients. Progression-free survival, which served as the primary endpoint of the patients, was defined from the date of initial histological diagnosis to the date of the first progression at any site or the last follow-up. Overall survival (OS) was the secondary endpoint of the patients and was defined from the initial histological diagnosis to the date of death from any cause or the last follow-up.

### Statistical Analysis

Statistical analyses were conducted using SPSS Statistics version 22.0 (IBM Corp., Chicago). Receiver operating characteristic (ROC) analyses were performed with PFS and OS as end-point, and areas under the curve (AUCs) of the ROCs were calculated and compared by Z test to evaluate the discriminatory ability of the metabolic PET parameters (14). Linear correlation analysis was conducted to evaluate the correlation among metabolic tumor burdens of different regional lesions. We applied the X-Tile, a clinically validated bio-informatics tool for biomarker evaluation and cut-point analyzing, to determine the cut-off values (14, 15). The survival curves were generated using Kaplan–Meier analysis and evaluated by log-rank test. Univariate and multivariate analyses were conducted by Cox regression model to determine the independent prognostic indicators. All the statistical analyses were considered significant at P <0.05.

## Results

### Patient Characteristics

The characteristics of the 76 patients enrolled in the study were summarized in **Table 1**. The cohort had 35 males (46.1%) and 41 females (53.9%), with the median age of 52 years (ranging from 19 to 80 years). Pre-treatment EBV-DNA test was performed in 28 patients with the positive rate of 82.1% (23 patients), and post-treatment EBV-DNA test was performed in 41 patients with the positive rate of 48.8% (20 patients). Twenty-one patients (27.6%) were in early stage (Stage I–II), and 55 patients (72.4%) were in advanced-stage (Stages III–IV). A total of 10 (13.2%), 32 (42.1%), 16 (21.0%), and 18 (23.7%) patients were staged at T1 to T4, and 24 (31.6%) and 52 (68.4%) patients were staged at N0–1 and N2–3, respectively. Distant metastasis (M1) was present in 22 patients (28.9%), including bone metastasis in 13 patients, adrenal gland metastasis in eight patients, liver metastasis in five patients, distant lymph-node metastasis in 14 patients, and parietal pleura/thoracic wall metastasis in three patients, and no patients had central nervous system metastasis. The treatment approach for operable disease was surgery in nine patients, surgery with chemotherapy in 23 patients, and surgery with chemo-radiotherapy in four patients. All the patients underwent surgery had R0 resection. For inoperable disease, 26 patients received chemotherapy, and 14 patients received chemo-radiotherapy.

**Table 1:** Patient characteristics.

	No. patients (n = 76)
Gender	
Male	35 (46.1%)
Female	41 (53.9%)
Age	
<52	37 (48.7%)
≥52	39 (51.3%)
T stage	
T1	10 (13.2%)
T2	32 (42.1%)
T3	16 (21.0%)
T4	18 (23.7%)
N stage	
N0–1	24 (31.6%)
N2–3	52 (68.4%)
M stage	
M0	54 (71.1%)
M1	22 (28.9%)
Tumor stage	
I–II	21 (27.6%)
III–IV	55 (72.4%)
Treatment method	
Surgery only	9 (11.8%)
Surgery + Chemotherapy	23 (30.3%)
Surgery + Chemotherapy + RT	4 (5.3%)
Palliative Chemotherapy	26 (34.2%)
Chemotherapy + RT	14 (18.4%)
Disease Progression	
Yes	34 (44.7%)
No	42 (55.3%)
Survival	
No	15 (19.7%)
Yes	61 (80.3%)

RT, radiotherapy.

### ROC Curve Analysis of PET Parameters in Pulmonary LELC

**Figure 2:**
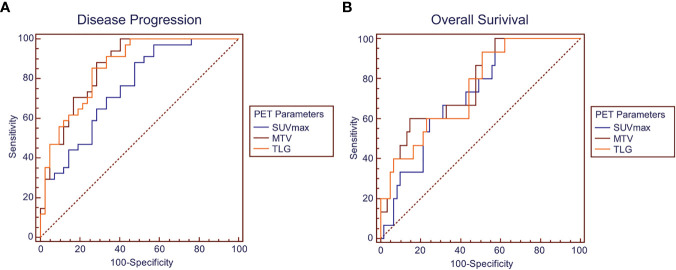
ROC curves showing discriminatory ability of SUVmax, MTV, and TLG for **(A)** disease progression and **(B)** overall survival.

ROC curves were generated for disease progression and overall survival to evaluate the discriminatory ability of the PET parameters (**Figure 2**). As shown in **Table 2**, the AUC was 0.857 (95% confidence interval (CI) 0.758–0.927; P < 0.001) for TLG, 0.862 (95% CI 0.764–0.930; P < 0.001) for MTV, and 0.754 (95% CI 0.641–0.845; P < 0.001) for SUVmax in the prediction of disease progression. The following Z test showed that TLG had significant higher AUC value than SUVmax (P = 0.041). MTV had higher AUC value than SUVmax, but the difference did not reach statistical significance (P = 0.056).

**Table 2:** Comparison of AUCs of PET parameters for disease progression and overall survival.

	AUC	95% CI	P-value^*^	P-value^†^
Disease Progression				
TLG	0.857	0.758–0.927	<0.001	0.042
MTV	0.862	0.764–0.930	<0.001	0.056
SUVmax	0.754	0.641–0.845	<0.001	–
Overall Survival				
TLG	0.751	0.638–0.843	<0.001	0.677
MTV	0.773	0.662–0.861	<0.001	0.507
SUVmax	0.720	0.605–0.817	<0.001	–

^*^P-values of ROC curve analysis. **^†^**Comparison of AUC values between SUVmax, TLG, and MTV using Z test method.

SUV, standard uptake value; MTV, metabolic tumor volume; TLG, total lesion glycolysis.

As for the discriminatory ability of overall survival, the AUC was 0.751 (95% CI 0.638–0.843; P < 0.001) for TLG, 0.773 (95% CI 0.662–0.861; P < 0.001) for MTV, and 0.720 (95% CI 0.605–0.817; P < 0.001) for SUVmax. No significant difference was found between the AUC values of the parameters by Z test.

### Prognostic Value of PET Parameters in Pulmonary LELC

The median follow-up duration was 33 months, ranging from 0.5 to 90 months, during which 34 patients (44.7%) had disease progression and 15 patients (19.7%) dead. Univariate analysis found that higher tumor stage (HR 4.60, 95% CI 2.28–9.29; P < 0.001), EBV-DNA copies (baseline: HR 3.29, 95% CI 1.13–9.62, P = 0.029; post-treatment: HR 3.03, 95% CI 1.20–7.61, P = 0.019), baseline NSE level (HR 2.48, 95% CI 1.23–4.98; P = 0.011), SUVmax (HR 2.10, 95% CI 1.07–4.13; P = 0.031), MTV (HR 4.18, 95% CI 2.11–8.30; P < 0.001) and TLG (HR 5.19, 95% CI 2.57–10.50; P < 0.001) were significantly correlated with worse PFS, and higher tumor stage (HR 9.84, 95% CI 3.07–31.51; P < 0.001), baseline NSE level (HR 4.08, 95% CI 1.32–12.58; P = 0.014), MTV (HR 4.54, 95% CI 1.61–12.82; P = 0.004) and TLG (HR 3.41, 95% CI 1.21–9.59; P = 0.020) were significantly correlated with worse OS.

**Figure 3:**
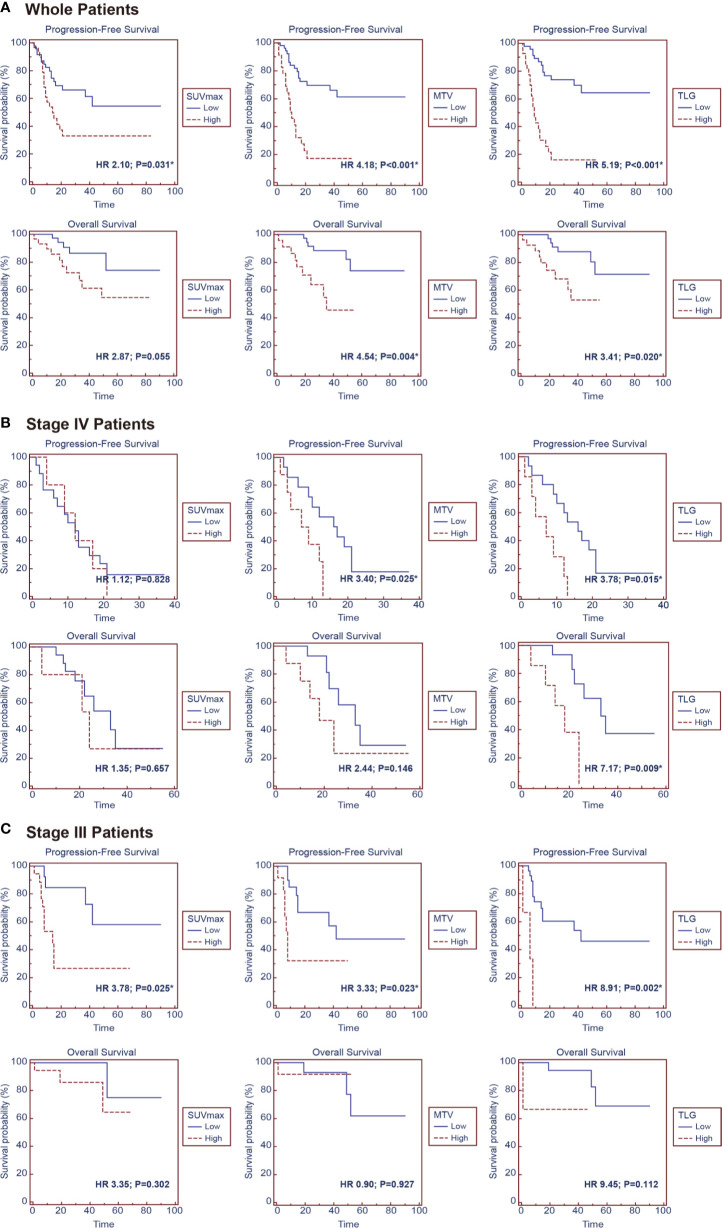
Kaplan–Meier survival curves for progression-free survival and overall survival according to SUVmax, MTV, and TLG in the groups of **(A)** whole patients, **(B)** stage IV patients and **(C)** stage III patients.

Patients in Stage 3 or Stage 4 were individually extracted for subgroup analysis, which found that higher MTV (HR3.40, 95% CI 1.17–9.90; P = 0.025) and TLG (HR 3.78, 95% CI 1.29–11.08; P = 0.015) were significantly correlated with worse PFS, and higher TLG (HR 7.17, 95% CI 1.63–31.49; P=0.009) was significantly correlated with worse OS in Stage 4 patients, meanwhile higher post-treatment EBV-DNA copy (HR 3.73, 95% CI 1.02–13.66; P = 0.045), SUVmax (HR3.78, 95% CI 1.18–12.15; P = 0.025), MTV (HR3.33, 95% CI 1.18–9.42; P = 0.023), and TLG (HR 8.91, 95% CI 2.18–36.26; P = 0.002) were significantly correlated with worse PFS in Stage 3 patients (**Figure 3** and **Table 3**).

**Table 3:** Univariate analysis for PFS and OS in patients with pulmonary LELC.

Variables	Progression-free survival			Overall survival		
	HR	95% CI	P-value	HR	95% CI	P-value
**Whole Patients**						
Age (≥52 *vs*. <52)	0.87	0.45–1.70	0.681	1.71	0.61–4.79	0.312
Gender (Male *vs*. Female)	1.03	0.53–2.02	0.931	0.93	0.34–2.57	0.886
Tumor stage (IV *vs*. I–III)	4.60	2.28–9.29	<0.001^*^	9.84	3.07–31.51	<0.001^*^
Pre-treatment EBV-DNA (≥85,500 *vs*. <85500)	3.29	1.13–9.62	0.029^*^	2.20	0.48–10.01	0.310
Post-treatment EBV-DNA (≥7,650 *vs*. <7,650)	3.03	1.20–7.61	0.019^*^	2.29	0.37–14.27	0.373
Pre-treatment lung cancer associated serum tumor biomarker (Normal *vs*. Abnormal)						
CA153	0.88	0.29–2.64	0.823	0.72	0.09–5.97	0.757
CEA	0.68	0.09–4.99	0.704	1.67	0.22–12.98	0.622
HCG-*β*	0.72	0.21–2.47	0.606	3.69	0.86–15.82	0.079
NSE	2.48	1.23–4.98	0.011^*^	4.08	1.32–12.58	0.014^*^
Post-treatment lung cancer associated serum tumor biomarker (Normal *vs*. Abnormal)						
CA153	1.46	0.54–3.93	0.452	0.66	0.08–5.72	0.708
CEA	1.03	0.24–4.37	0.973	3.88	0.78–19.34	0.098
HCG-*β*	0.32	0.05–2.46	0.275	1.43	0.15–13.81	0.758
NSE	1.68	0.68–4.15	0.260	3.77	0.82–17.22	0.087
PET Parameters						
SUVmax (≥17.5 *vs*. <17.5)	2.10	1.07–4.13	0.031^*^	2.87	0.98–8.42	0.055
MTV (≥200 *vs*. <200)	4.18	2.11–8.30	<0.001^*^	4.54	1.61–12.82	0.004^*^
TLG (≥1,000 *vs*. <1,000)	5.19	2.57–10.50	<0.001^*^	3.41	1.21–9.59	0.020^*^
**Stage IV patients**						
Pre-treatment EBV-DNA (≥22,000 *vs*. <22,000)	1.19	0.28–5.01	0.810	0.42	0.07–2.59	0.349
Post-treatment EBV-DNA (≥2,000 *vs*. <2,000)	1.69	0.45–6.33	0.435	1.49	0.20–11.06	0.694
Pre-treatment NSE (Normal *vs*. Abnormal)	2.62	0.91–7.51	0.074	1.66	0.41–6.74	0.482
PET Parameters						
SUVmax (≥20.0 *vs*. <20.0)	1.12	0.40–3.12	0.828	1.35	0.36–5.13	0.657
MTV (≥350 *vs*. <350)	3.40	1.17–9.90	0.025^*^	2.44	0.73–8.14	0.146
TLG (≥2,250 *vs*. <2250)	3.78	1.29–11.08	0.015^*^	7.17	1.63–31.49	0.009^*^
**Stage III patients**						
Pre-treatment EBV-DNA (≥10,000 *vs*. <10,000)	1.62	0.32–8.15	0.558	not available**^†^**		0.443
Post-treatment EBV-DNA (≥1,600 *vs*. <1,600)	3.73	1.02–13.66	0.045^*^	not available**^†^**		0.725
Pre-treatment NSE (Normal *vs*. Abnormal)	2.71	0.91–8.09	0.073	not available**^†^**		0.480
PET Parameters						
SUVmax (≥15.5 *vs*. <15.5)	3.78	1.18–12.15	0.025^*^	3.35	0.34–33.24	0.302
MTV (≥125 *vs*. <125)	3.33	1.18–9.42	0.023^*^	0.90	0.09–8.86	0.927
TLG (≥1,830 *vs*. <1,830)	8.91	2.18–36.26	0.002^*^	9.45	0.59–151.82	0.112

^*^P < 0.05; **^†^**Unavailable HR value and wide range of 95% CI due to limited events in one group.

PFS, progression-free survival; OS, overall survival; SUV, standard uptake value; MTV, metabolic tumor volume; TLG, total lesion glycolysis.

As PET parameters of SUV, MTV, and TLG had intrinsic connections; the parameters were taken into individual multivariate regression models to conduct analysis. The multivariate analysis showed that higher MTV (P = 0.026) and TLG (P = 0.019) were independent prognostic factor for worse PFS. In subgroup analysis, higher TLG (P = 0.042) was identified as an independent prognostic factor for worse PFS in Stage 4 patients (**Table 4**).

**Table 4:** Multivariate analysis for PFS and OS in patients with pulmonary LELC.

Variables	P value for PFS			P value for OS		
	Model 1	Model 2	Model 3	Model 1	Model 2	Model 3
**Whole Patients**						
Pre-treatment EBV-DNA	0.410	0.949	0.749	0.538	0.700	0.588
Post-treatment EBV-DNA	0.096	0.121	0.217	0.187	0.155	0.267
Tumor Stage	0.088	0.943	0.884	0.212	0.362	0.399
SUVmax	0.074			0.278		
MTV		0.026^*^			0.904	
TLG			0.019^*^			0.378
**Stage IV patients**						
Pre-treatment EBV-DNA	0.232	0.881	0.849	0.342	0.661	0.787
Post-treatment EBV-DNA	0.199	0.259	0.396	0.390	0.284	0.325
SUVmax	0.090			0.334		
MTV		0.087			0.607	
TLG			0.042^*^			0.436
**Stage III patients**						
Pre-treatment EBV-DNA	0.413	0.474	0.436	0.674	0.896	0.773
Post-treatment EBV-DNA	0.227	0.235	0.204	0.670	0.903	0.771
SUVmax	0.814			0.979		
MTV		0.130			0.736	
TLG			0.250			0.972

^*^P < 0.05.

PFS, progression-free survival; OS, overall survival; SUV, standard uptake value; MTV, metabolic tumor volume; TLG, total lesion glycolysis.

### Linear Correlation Analysis of Different Regional Lesions

**Figure 4:**
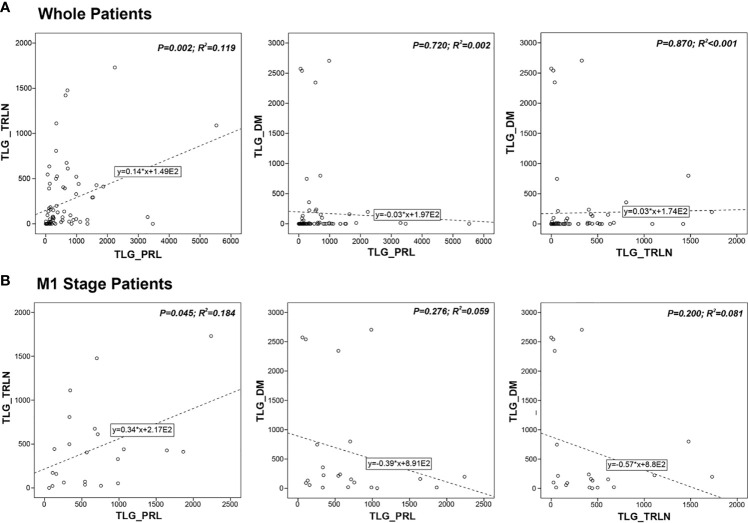
Correlations between metabolic tumor burdens of primary lesion (PRL), thoracic lymph-node lesion (TRLN) and distant metastasis (DM) in **(A)** whole patients’ group and **(B)** M1 stage patients’ group. Linear correlation was found between TLG_-TRLN_ and TLG_-PRL_, whereas TLG_-DM_ did not show significant correlation with TLG_-PRL_ and TLG_-TRLN_.

Linear correlation analysis was done to investigate the correlation between the different regional lesions. As **Figure 4A** shows, linear correlation was found between TLG_-TRLN_ and TLG_-PRL_ (r^2^ = 0.119, P = 0.002), whereas TLG_-DM_ did not show significant correlation with TLG_-PRL_ (r^2^ = 0.002, P = 0.720) and TLG_-TRLN_ (r^2^ < 0.001, P = 0.870). The linear correlation analysis for M1 stage patients generated similar results. TLG_-TRLN_ linear correlated with TLG_-PRL_ (r^2^ = 0.184, P = 0.045), whereas TLG_-DM_ did not show significant correlation with TLG_-PRL_ (r^2^ = 0.059, P = 0.276) and TLG_-TRLN_ (r^2^ = 0.081, P = 0.200) (**Figure 4B**). Similar results were also observed when the analysis was done for MTV.

### Prognostic Significance of Regional PET Parameters in Pulmonary LELC

Multivariate analysis was conducted for PET parameters of the different regional lesions to determine their prognostic significance individually. The results showed that TLG_-PRL_ (P = 0.002 for PFS and P = 0.023 for OS) and TLG_-DM_ (P = 0.001 for PFS, and P = 0.005 for OS) were significant independent prognostic indicators for PFS and OS in pulmonary LELC patients, whereas TLG_-TRLN_ was not found to be independent prognostic indicator. Similarly, MTV_-PRL_ (P = 0.002 for PFS, and P = 0.003 for OS) and MTV_-DM_ (P = 0.003 for PFS, and P = 0.019 for OS) were identified to be significant independent prognostic indicators for PFS and OS, whereas MTV_-TRLN_ was not. None of the SUVmax values of the different regional lesions was found to be independent prognostic indicator for PFS and OS (**Table 5**).

**Table 5:** Multivariate analysis of PET parameters derived from different regional lesions.

	P value for PFS	P value for OS
**Model 1**		
SUVmax_-PRL_	0.212	0.524
SUVmax_-TRLN_	0.674	0.069
SUVmax_-DM_	0.801	0.146
**Model 2**		
MTV_-PRL_	0.002^*^	0.003^*^
MTV_-TRLN_	0.469	0.631
MTV_-DM_	0.003^*^	0.019^*^
**Model 3**		
TLG_-PRL_	0.002^*^	0.023^*^
TLG_-TRLN_	0.608	0.505
TLG_-DM_	0.001^*^	0.005^*^

^*^P < 0.05.

PFS, progression-free survival; OS, overall survival; PRL, primary tumor lesion; TRLN, thoracic lymph-node lesion; DM, distant metastasis; SUV, standard uptake value; MTV, metabolic tumor volume; TLG, total lesion glycolysis.

## Discussion

Pulmonary LELC has been recognized as a unique lung carcinoma which is closely related to EBV infection with genomic landscape and clinicopathological features strikingly distinctive to NSCLC (16). Pulmonary LELC had better prognosis, and previous studies also have discovered that classic lung cancer oncogenic drivers including KRAS, EGFR, BRAF, ALK, and ROS1 were limitedly involved in tumorigenesis and progression of pulmonary LELC (8, 17). However, due to its rarity, standard clinical guideline for pulmonary LELC has not been established. To our knowledge, the current study firstly investigated the value of PET parameters in pulmonary LELC patients and demonstrated the independent prognostic significance of MTV and TLG. In addition, the study individually analyzed different regional lesions of whole-body tumor burden of pulmonary LELC and discovered that tumor burdens of primary lesion and distant metastasis were the main contributor to disease progression and worse survival of the patients.

ROC curve analysis of the PET parameters for the patient’s outcome suggested significant discriminatory ability of SUVmax, MTV, and TLG in predicting disease progression and survival with all the AUC values over 0.72. Generally, AUC values of a risk prediction model over 0.70 are considered to be useful, with values over 0.80 considered to be excellent (18), which indicated considerable prediction significance of the PET parameters in pulmonary LELC patients, especially for the TLG and MTV in predicting disease progression (AUC value 0.86). The following Z test additionally demonstrated significantly higher discriminatory ability of MTV and TLG than SUVmax, which suggested the potential superiority of volumetric parameters than SUVmax in pulmonary LELC patients. In addition, although all the PET parameters were found to be significantly associated with worse survival in the univariate analysis, the HR values of MTV and TLG were higher than that of SUVmax, which were further identified to be independent prognostic factors for PFS whereas SUVmax was not. The findings were consistent with previous studies which also failed to demonstrate SUV as an independent prognostic indicator (19, 20).

SUVmax has been widely utilized because of its convenience in measuring; however, as it only represented the highest metabolic intensity, bias could be generated especially when assessing patients with multiple lesions. Volumetric parameters including MTV and TLG take tumor volume into metabolism calculation, which represent a combination of metabolic features and morphological findings and could be considered as an expression of tumor burden and aggressiveness (21, 22). MTV and TLG could be more precise in reflecting real tumor burden of pulmonary LELC and led to a more accurate prognosis evaluation, which was consistent with their demonstrated prognostic role in other malignancies including lymphoma, gastrointestinal stromal tumor and pancreatic cancer (21–23). On the other hand, the clinical application of the volumetric PET parameters may still be immature due to the lack of standardized methodology for measurement, and a wide-range of cutoff values was reported in the literature under different measurement methods, which called for unification and standardization in the future.

Although our previous study demonstrated that chemotherapy with gemcitabine and cisplatin could serve as the first-line treatment regimen for pulmonary LELC, which achieved the highest response rate and longest PFS compared with other regimens (9), the standard clinical management protocol for pulmonary LELC has not been established. Comprehensive treatment schemes are needed to be formed with the participation of medical oncologist, thoracic surgeon, and thoracic radiologist, and taking our findings into consideration, the corresponding role of chemotherapy, surgery and radiotherapy should be further clarified and evaluated.

In the study, we found that the tumor burden of TRLN significantly correlated with PRL, whereas the extent of DM did not show significant correlation with PRL or TRLN. In clinic, we did notice the lesion distribution pattern varied when checking PET images that either were relatively small PRL with large extent of DM or the opposite distribution pattern existed in our cohort (**Figure 1**). The findings indicated that the extent of TRLN metastasis was significantly dependent on the local progression of primary tumor, whereas the distribution of distant metastatic lesions conformed to the parallel progression models (24). The non-colinearity between the different regional lesions raised the question as to which lesion dominated the patient’s outcome, and the medical controlling of which lesion would be the most efficient in improving prognosis.

It is interesting in our study that the tumor burden of PRL or DM was independent prognostic indicator for worse PFS and OS, whereas the tumor burden of TRLN was not. Oh et al. once classified whole-body tumor lesions of NSCLC patients into thoracic and extra-thoracic tumor burden and found that the extra-thoracic rather than thoracic tumor burden significantly correlated with survival (25). In line with that study, our findings highlighted the considerable influence of DM tumor burden on patient’s outcomes. Nevertheless, concerning further dividing thoracic tumor burden into PRL and TRLN for investigating their significance individually, little has been investigated, and we found that the PRL had a more important role in influencing survival.

One possible explanation for the discrepancy of the impact on survival could be the difference in chemosensitivity between TRLN and other lesions. Numerous basic studies have revealed diverse gene expression patterns of the different lesions and observed that lymph-node metastasis was often biologically less malignant (26–28). Primary and distant metastatic lesions were more heterogeneous in cell clonality and tumor microenvironment, which contribute to more tough characteristics in drug delivery, hypoxia, and immune escape, thus more resistant to chemotherapy (29, 30). The lesions were not merely different in anatomical sites, but also in biological and histological properties. For clinical studies, Makino et al. (31) and Alita et al. (32) observed significantly lower metabolic intensity of lymph-node lesions after neo-adjuvant chemotherapy, which suggested better treatment response of lymph-node metastasis compared with other lesions. In the current study, chemotherapy was routinely given to those with lymph-node or distant metastases; assuming that TRLN lesions could be better controlled by chemotherapy regimens, it could explain that it was PRL and DM tumor burdens that remained the main contributor to the patients’ worse prognosis and ultimately be identified as independent prognostic indicators for PFS and OS.

Based on the findings, clinical management for PRL and DM lesions should be paid enough attention in treating pulmonary LELC patients, which required multi-disciplinary cooperation. Compared with numerous TRLN lesions that were located in the central of mediastinal vessels, pulmonary lobes, esophagus, trachea and heart, peripheral PRL or DM was actually more suitable to be resected by surgery or the target for radiotherapy, and thus the management disciplines should be formulated by medical oncologist, thoracic surgeon, and thoracic radiologist based on individual conditions of each patient. In addition, still as a hypothesis, the similarities between pulmonary LELC and NPC could generate a speculation concerning the role of radiotherapy in treating pulmonary LELC, which required further exploitation by future studies. Both of the diseases were closely related to EBV infection, and the histopathological manifestation between pulmonary LELC and pulmonary metastatic NPC is so similar that pathological diagnosis of pulmonary LELC requires exclusion of primary NPC by nasopharyngoscopy. In addition, pulmonary LELC is more sensitive to gemcitabine-based chemotherapy, which is also similar to NPC rather than NSCLC. Overall, more active utilization of multi-disciplinary cooperation in treating pulmonary LELC patients should be considered, and is worthy to be further investigated by random controlled clinical trials.

Overall, ^18^F-FDG PET/CT has a considerable value in distinguishing pulmonary LELC patients with higher risk of adverse events and worse prognosis, knowing the tendency could assist in clinical decision-making during follow-up. A more rigorous follow-up protocol with shorter intervals could be applied to these patients, and more active interventions could be considered when suspected lesions of metastasis or local recurrence were found. With PET/CT identifying high-risk groups and monitoring the disease status during follow-up and the early intervention of recurrent disease under treatment schemes incorporating different modalities, the patients’ prognosis is expected to be further improved.

We also need to mention that we failed to identify the independent prognostic significance of tumor stage in the multivariate analysis, which could be due to the low number of patients in early stage that decreased the statistical efficacy of analysis for this parameter. The relatively limited total patient number due to rarity of the disease could be another reason. The prognostic value of tumor stage is needed to be further verified by future studies incorporating a larger group of patients.

A potential limitation of the current study is the relatively small group of patients as mentioned above. Although we included the largest scale of pulmonary LELC patients who underwent PET/CT examination to date, the statistical efficacy could still be influenced. Secondly, we used the 7^th^ AJCC staging system in the study which was not the latest 8^th^ edition, mainly due to the fact that most of the included patients were treated before 2016, and the current re-staging would be inaccurate in some earlier treated patients with incomplete data and bring bias to the study. Thirdly, the detection of EBV-DNA copy number and serum tumor biomarkers was not a clinical routine, which caused missing data in some patients. Besides, all the included patients were of Asian descent, which may limit the generalizability to other populations. In addition, we included patients with PET examinations conducted on two different scanners. As the PET parameters are influenced by several aspects including patient-features, blood glucose levels, and imaging technical characteristics, potential bias could be generated when comparing the PET parameters from different scanners, and thus our findings should be interpreted with caution and further validated.

In conclusion, the study demonstrated that metabolic PET parameters of MTV and TLG had independent prognostic significance for pulmonary LELC patients, with a satisfactory discriminatory ability for treatment outcomes superior to SUVmax. Besides, non-colinearity was found among the metabolic volume of different regional lesions. PET-derived metabolic burdens of primary lesion and distant metastasis were independently correlated with unfavorable outcomes, whereas those of thoracic lymph-node lesions were not. The findings supported the incorporation of ^18^F-FDG PET into clinical treatment protocols for pulmonary LELC and implied multi-disciplinary cooperation especially for primary and distant metastatic lesions to further improve prognosis of pulmonary LELC patients.

## Data Availability Statement

The raw data supporting the conclusions of this article will be made available by the authors. Further inquiries can be directed to the corresponding authors.

## Ethics Statement

All procedures performed in studies involving human participants were in accordance with the ethical standards of the institutional and/or national research committee and with the 1964 Declaration of Helsinki and its later amendments or comparable ethical standards.

## Author Contributions

YL and WF designed the study. WZ, HZ, and JH contributed to the literature review, data collection, data analysis, and drafting. ZC and XZ were involved in data acquisition and technical support. All authors critically reviewed and approved the final manuscript. YL, WF, and XZ are responsible for the integrity of the work as a whole. All authors contributed to the article and approved the submitted version.

## Conflict of Interest

The authors declare that the research was conducted in the absence of any commercial or financial relationships that could be construed as a potential conflict of interest.
